
JOURNAL INFORMATION
==============================
NLM Title Abbreviation: J High Energy Phys
Journal ID: J High Energy Phys
NLM Journal ID: 101668727

Journal of High Energy Physics

ISSN: 1126-6708
EISSN: 1029-8479

ARTICLE INFORMATION
==============================
PMCID: PMC8827111
PMID: 35226715
DOI: 10.1007/JHEP02(2022)033
Article ID: 17718
Article version: 1
Subjects: Erratum

Erratum to: Anomaly free Froggatt-Nielsen models of flavor

http://orcid.org/0000-0003-0798-9512 Smolkovič Aleks aleks.smolkovic@ijs.si
1
Tammaro Michele tammarme@mail.uc.edu
2
Zupan Jure zupanje@ucmail.uc.edu
2
1 grid.11375.31 0000 0001 0706 0012 Jožef Stefan Institute, Jamova 39, 1000 Ljubljana, Slovenia
2 grid.24827.3b 0000 0001 2179 9593 Department of Physics, University of Cincinnati, Cincinnati, Ohio 45221 USA
Electronic publication date: 2022 Feb 4
Print publication date: 2022
Volume: 2022
Issue: 2
Electronic Location ID: 33
Received 2021 Oct 15; Accepted 2022 Jan 16
Copyright: © The Author(s) 2022
License: Open Access. This article is distributed under the terms of the Creative Commons Attribution License (CC-BY 4.0), which permits any use, distribution and reproduction in any medium, provided the original author(s) and source are credited.
License URL: https://creativecommons.org/licenses/by/4.0/

Erratum for: 10.1007/JHEP10(2019)188
issue-copyright-statement: © The Author(s) 2022

==============================
Erratum to: JHEP10(2019)188

ArXiv ePrint: 1907.10063

The online version of the original article can be found at 10.1007/JHEP10(2019)188

Publisher’s Note

Springer Nature remains neutral with regard to jurisdictional claims in published maps and institutional affiliations.


References

[1] Smolkovič A Tammaro M Zupan J Anomaly free Froggatt-Nielsen models of flavor JHEP 2019 10 188 10.1007/JHEP10(2019)188 PMC8827111 35226715
[2] C. Bourrely, I. Caprini and L. Lellouch, Model-independent description of B → πlv decays and a determination of |V(ub)|, Phys. Rev. D 79 (2009) 013008 [Erratum ibid. 82 (2010) 099902] [arXiv:0807.2722] [INSPIRE].
[3] Particle Data Group collaboration, Review of Particle Physics, PTEP 2020 (2020) 083C01 [INSPIRE].
[4] Y. Aoki et al., FLAG Review 2021, arXiv:2111.09849 [INSPIRE].
[5] Na H Davies CTH Follana E Lepage GP Shigemitsu J The D → K, lv Semileptonic Decay Scalar Form Factor and |Vcs| from Lattice QCD Phys. Rev. D 2010 82 114506 10.1103/PhysRevD.82.114506
[6] H. Na, C.T.H. Davies, E. Follana, J. Koponen, G.P. Lepage and J. Shigemitsu, D → π, lv Semileptonic Decays, |V cd| and 2 nd Row Unitarity from Lattice QCD, Phys. Rev. D 84 (2011) 114505 [arXiv:1109.1501] [INSPIRE].
[7] ETM collaboration, Scalar and vector form factors of D → π(K)ℓν decays with N f = 2 + 1 + 1 twisted fermions, Phys. Rev. D 96 (2017) 054514 [Erratum ibid. 99 (2019) 099902] [Erratum ibid. 100 (2019) 079901] [arXiv:1706.03017] [INSPIRE].
[8] HPQCD and HPQCD collaborations, Improved Vcs determination using precise lattice QCD form factors for D → Kℓν, Phys. Rev. D 104 (2021) 034505 [arXiv:2104.09883] [INSPIRE].
[9] E. Dalgic, A. Gray, M. Wingate, C.T.H. Davies, G.P. Lepage and J. Shigemitsu, B meson semileptonic form-factors from unquenched lattice QCD, Phys. Rev. D 73 (2006) 074502 [Erratum ibid. 75 (2007) 119906] [hep-lat/0601021] [INSPIRE].
[10] Fermilab Lattice and MILC collaborations, |V ub | from B → πℓν decays and (2+1)-flavor lattice QCD, Phys. Rev. D 92 (2015) 014024 [arXiv:1503.07839] [INSPIRE].
[11] J.M. Flynn et al., B → πℓν and B s → Kℓν form factors and |V ub | from 2+1-flavor lattice QCD with domain-wall light quarks and relativistic heavy quarks, Phys. Rev. D 91 (2015) 074510 [arXiv:1501.05373] [INSPIRE].
[12] HPQCD collaboration, Rare decay B → Kℓ + ℓ − form factors from lattice QCD, Phys. Rev. D 88 (2013) 054509 [Erratum ibid. 88 (2013) 079901] [arXiv:1306.2384] [INSPIRE].
[13] J.A. Bailey et al., B → Kl + l − Decay Form Factors from Three-Flavor Lattice QCD, Phys. Rev. D 93 (2016) 025026 [arXiv:1509.06235] [INSPIRE].
